# Acute inferior ST-elevation myocardial infarction due to delirium tremens: a case report

**DOI:** 10.1186/s13256-019-2246-x

**Published:** 2019-10-09

**Authors:** Maxwell D. Mirande, George Kubac, Anh T. Nguyen

**Affiliations:** 1 Western University of Health Sciences College of Osteopathic Medicine of the Pacific-Northwest, 200 Mullins Drive, Lebanon, OR 97355 USA; 2 Sky Lakes Medical Center, 2865 Daggett Avenue, Klamath Falls, 97601 OR USA

**Keywords:** Delirium tremens, Alcohol withdrawal syndrome, Acute coronary events, ST-elevation myocardial infarction, STEMI, Catecholamine-associated myocardial injury

## Abstract

**Background:**

Delirium tremens is a severe form of alcohol withdrawal syndrome. Literature documenting acute coronary events in the setting of alcohol withdrawal remains scarce. An accepted hypothesis for the underlying process is focused on the hyperadrenergic state that leads to coronary vasospasm and increased myocardial oxygen demand.

**Case presentation:**

A 47-year-old Caucasian man with a past medical history of tobacco and alcohol abuse, hypertension, and anxiety presented to the emergency department for crampy epigastric abdominal pain with intractable nausea and vomiting for the past 2 days. His reported last alcoholic intake was about 10 days prior; however, outpatient records indicated otherwise. He was admitted for electrolyte replacement and fluid resuscitation secondary to gastrointestinal losses from presumed early alcohol withdrawal syndrome. The following night, he developed acute substernal chest pain with elevated cardiac enzymes. Electrocardiography showed an acute inferoposterior infarct with reciprocal changes in leads V1–V4. The patient was taken for emergent catheterization, and a drug-eluting stent was placed in the middle of the left anterior descending artery. Postcatheterization electrocardiography showed sustained inferolateral ST elevations consistent with acute injury pattern. The patient had not required any benzodiazepines until this point. On the morning of catheterization, the patient’s Clinical Institute Withdrawal Assessment for Alcohol–Revised score was 19 with a high of 25, and he was actively hallucinating. He was treated for delirium tremens and an acute coronary event along with an incidental pneumonia. He did not require any benzodiazepines during the last 4 days of admission, and he made a full recovery.

**Conclusions:**

The prevalence of alcohol dependence in hospitalized patients is substantial. Although our patient was being treated with the standard protocols for alcohol withdrawal, he rapidly developed delirium tremens, which led to an acute inferior ST-elevation myocardial infarction in the setting of nonoccluded coronary vessels. This case report adds to the sparse literature documenting acute coronary events in the setting of alcohol withdrawal and suggests that our patient’s ST-elevation myocardial infarction is not fully explained by the current coronary vasospasm hypothesis, but rather was in part the result of direct catecholamine-associated myocardial injury. Further research should be conducted on prophylactic agents such as β-blockers and calcium channel blockers.

## Background

Delirium tremens (DT) is a severe form of alcohol withdrawal syndrome that typically presents within 48–96 hours and is defined by delirium, hallucinations, seizures, autonomic hyperactivity, and psychomotor disturbances [1]. There have been a few cases documenting the association between alcohol withdrawal, ST-segment changes, and acute coronary events; however, the literature on this topic remains limited [2–5].

The current hypothesis for the underlying process is the hyperadrenergic state that occurs during alcohol withdrawal leads to coronary vasospasm and increased myocardial oxygen demand [2, 3, 5, 6]. It has also been shown that patients with chronic alcoholism have intramyocardial small-vessel changes, such as vascular wall edema and perivascular fibrosis, that restrict the ability of the arteries to dilate during intense vasoconstriction [7]. In addition, thrombosis from enhanced platelet reactivity that occurs during alcohol withdrawal contributes to increased cardiovascular risk [8]. We present a case of a patient with an acute inferior ST-elevation myocardial infarction (STEMI) that occurred in the setting of DT that was complicated by community-acquired pneumonia (CAP). This case report adds to the scarce literature documenting the association between alcohol withdrawal and acute coronary events and brings into question the underlying pathophysiology.

## Case presentation

A 47-year-old Caucasian man with a past medical history of tobacco use, alcohol abuse, hypertension, and anxiety presented to the emergency department for crampy epigastric abdominal pain with intractable nausea and vomiting for the past 2 days. The patient also admitted to a chronic productive cough. The patient was brought in via emergency medical services after being orthostatic and tachycardic at the clinic earlier that day. The patient reported that his last alcoholic intake was about 10 days ago. However, outpatient records stated that the patient’s last drink could have been anywhere from 1 week to 3 days prior. The patient was drinking about 1 pint of vodka per day and stated that he had undergone inpatient alcohol detoxification five or six times in the past.

His vital signs upon admission showed a blood pressure of 158/111 mmHg, pulse rate of 115 beats/minute, temperature of 37.2 °C (99 °F), respiration of 20 breaths/minute, and a peripheral capillary oxygen saturation of 99%. His physical examination showed mild epigastric tenderness to palpation but was otherwise unremarkable. Workup included a chest x-ray, which showed few air bronchograms projecting into the posterior lung base, procalcitonin of 0.61 ng/ml, and lactate of 1.8 mmol/L. The patient’s sodium was 128 mmol/L, potassium 2.34 mmol/L, chloride 74.3 mmol/L, magnesium 1.2 mg/dl, glucose 202 mg/dl, and serum alcohol < 0.010 (g/dL). Laboratory tests showed a platelet count of 87 × 10^9^/L and mild chronic transaminitis. He was admitted to the hospital for electrolyte replacement and fluid resuscitation secondary to gastrointestinal losses from presumed early alcohol withdrawal syndrome. Urine *Streptococcus pneumoniae* and *Legionella* antigen tests were ordered at that time. The patient was started on 1 L of sodium chloride 0.9%, oral potassium chloride 20 mEq twice daily, daily banana bags (1 L of sodium chloride 0.9% with thiamine 100 mg, folic acid 1 mg, multivitamin for infusion 1 ampule, and magnesium sulfate 3 g), home metoprolol 25 mg twice per day, Clinical Institute Withdrawal Assessment for Alcohol–Revised (CIWA-Ar) checks, seizure precautions, and ampicillin-sulbactam for suspected pneumonia.

**Fig. 1:**
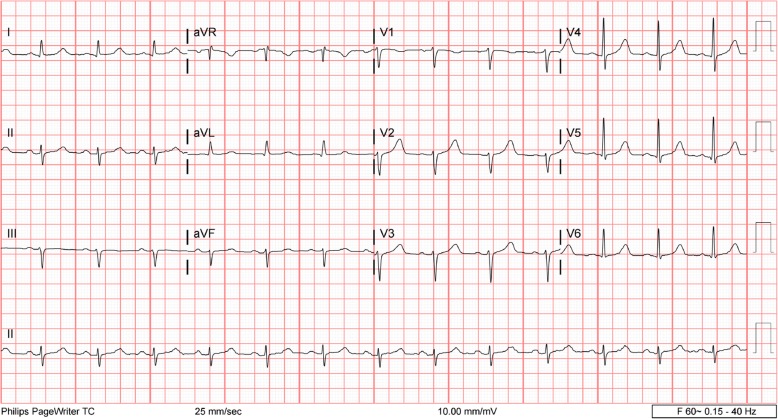
Electrocardiography performed the morning after admission showed sinus rhythm with left-axis deviation and poor R-wave progression

**Fig. 2:**
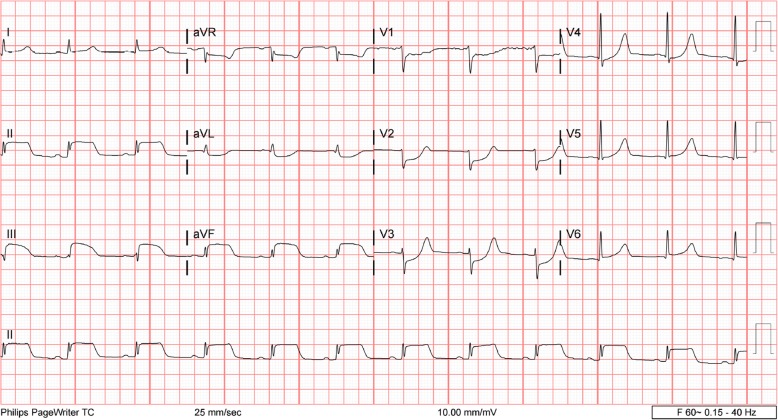
Electrocardiography performed during an episode of substernal chest pain showed acute inferoposterior infarct with reciprocal changes in leads V1–V4

**Fig. 3:**
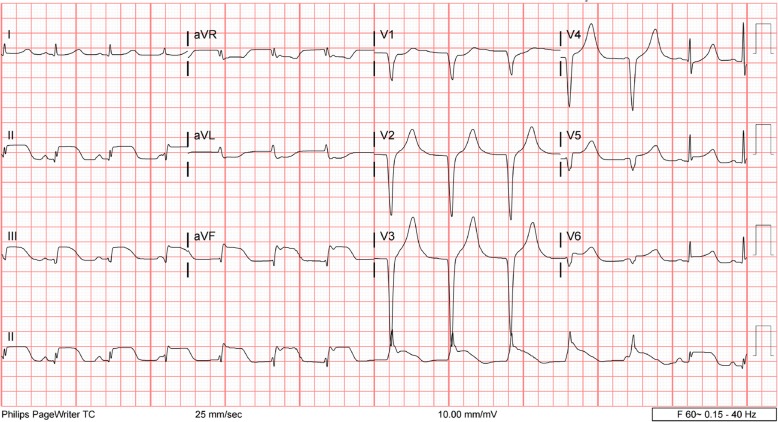
Electrocardiography performed after catheterization showed sinus rhythm with periods of accelerated idioventricular rhythm, inferolateral ST elevations consistent with acute injury pattern, and prolonged QT interval

The following day, the patient’s electrolyte imbalances had improved. Electrocardiography (ECG) showed sinus rhythm with left-axis deviation and poor R-wave progression (Fig. 1). The patient stated that he was feeling better and able to tolerate oral intake. He continued to receive antibiotics, sodium chloride 0.9% at 100 ml/hour, potassium chloride, and banana bags. At around 0130 hours the following morning, the patient became more irritable and reported acute substernal chest pain while sitting in bed. An ECG was obtained and showed an acute inferoposterior infarct with reciprocal changes in leads V1–V4 (Fig. 2). An urgent troponin measurement was 0.34 ng/ml (normal range, 0.00 to < 0.01 ng/ml). The patient was taken for emergent catheterization, and a 3.5 × 18-mm drug-eluting stent was placed in the middle of the left anterior descending artery (LAD). A postcatheterization ECG showed sinus rhythm with periods of accelerated idioventricular rhythm, inferolateral ST elevations consistent with acute injury pattern, and prolonged QT interval (Fig. 3).

Up to this point, the patient’s CIWA-Ar scores were 0. On the morning of catheterization, his CIWA-Ar score was recorded as 19 with a high of 25. The patient was actively hallucinating and required lorazepam, additional banana bags, and a 1:1 sitter. The patient did not develop seizures during this event. The patient was treated for an acute coronary event. A subsequent echocardiogram showed mild to moderate inferior hypokinesis at the base as well as anteroseptal and mild anterior hypokinesis with an ejection fraction of 50–55%. The patient developed atrial fibrillation with rapid ventricular response in the setting of his acute STEMI. The patient had a normal thyroid-stimulating hormone level and was rate-controlled with amiodarone and carvedilol. He was initially started on a statin, but this was discontinued after an unacceptable elevation of his liver function tests.

The patient’s *S. pneumoniae* antigen was positive the day following his acute STEMI, and his antibiotics were changed to intravenous ceftriaxone. He remained afebrile and never required supplemental oxygen. He made a full recovery and did not require any benzodiazepines during the last 4 days of admission. He stated that he was not interested in going back to rehabilitation and would prefer to quit on his own. The patient was discharged to home on day 7 with 10 days of amoxicillin-clavulanic acid, aspirin, clopidogrel, amiodarone, carvedilol, and Veteran Affairs follow-up.

## Discussion

Anginal chest pain generally occurs only when there is greater than 70% stenosis of a major coronary vessel. Our patient denied any prior chest pain symptoms before his acute STEMI. Although he patient had many risk factors for developing an acute cardiac event, including incidental pneumococcal pneumonia, he had no known coronary artery disease. His elevated troponin level and his ECG suggested an inferoposterior infarct due to a right coronary artery (RCA) occlusion because approximately 85% of the population has right-sided dominance [9]. However, the patient’s catheterization showed a small, normal, nondominant RCA and a normal, dominant left circumflex artery. This indicated that he had left dominance with patent vessels. The catheterization also revealed a large LAD that wrapped around the apex with 20–30% stenosis proximally and 70–80% stenosis in the middle portion. The vessel exhibited brisk Thrombolysis in Myocardial Infarction 3 flow.

Although the patient’s coronary vessels showed stenosis, there was no occlusion to explain his inferior STEMI. In this setting, there are several mechanisms that may explain what occurred. The patient could have had a thrombosis that occluded his coronary vessels and then spontaneously lysed prior to catheterization. Because there was no evidence of thrombosis or plaque rupture, this is unlikely. Although the current hypothesis of coronary spasm due to increased sympathetic hyperactivity remains a possible explanation, there was no documented vasospasm observed during catheterization. Additionally, the postcatheterization ECG showed sustained ST-segment elevations despite normal coronary flow (Fig. 3). These ST-segment elevations would have been expected to resolve almost instantly after stenting if the patient’s inferior STEMI were solely the result of reduced coronary flow. Furthermore, the lateral wall involvement seen on ECG in leads V5 and V6 suggests myocardial injury beyond the distribution of the LAD, which is not fully explained by a reduced flow state caused by vasospasm (Figs. 2 and 3). Hence, an alternative explanation that is more consistent with the clinical picture is the diffusely elevated circulating catecholamines during DT might have caused myocardial stunning or induced direct myocardial toxicity mimicking an occlusive coronary event. This is similar to one of the proposed hypotheses for stress cardiomyopathy, also known as *Takotsubo syndrome* [10]. The patient’s sustained ST-segment elevations after stenting and the apical wall motion abnormalities seen on an echocardiogram in the setting of nonoccluded coronary vessels are consistent with this pathophysiology. Also, the patient’s ECG at discharge returned to baseline with no observable Q waves or signs of permanent myocardial injury, which further supports in part a direct catecholamine-associated myocardial injury mechanism. Unfortunately, a follow-up echocardiogram was not obtained to confirm complete recovery of the wall motion abnormalities.

Because DT is an uncommon occurrence with current protocols, it is worth discussing how it might have developed in our patient. Our patient presented to the emergency department with intractable nausea and vomiting for 2 days. Although the patient’s reported last alcoholic intake was questionable, his presenting gastrointestinal symptoms and laboratory test results were consistent with the mild symptoms of alcohol withdrawal syndrome. The patient’s laboratory test results and clinical state were improving with initial resuscitation. He maintained CIWA-Ar scores of 0 and required no benzodiazepines within the first 24 hours. It was difficult to identify the exact timeline of when the patient developed DT and the STEMI because they seemed to occur simultaneously. As the patient’s cardiac issues were given priority, the first rising CIWA-Ar score was not recorded until after catheterization, when the patient required benzodiazepines. However, nursing staff reported that the patient became more irritable and was ripping off his bed covers, looking for his “key fob” in reference to his call light, prior to developing chest pain, suggesting that his delirium preceded his cardiac event.

DT typically does not occur suddenly; rather, it progresses in a more sequential timeline [1]. Our patient’s clinical course was complicated by incidental CAP. Although our patient remained afebrile and never developed respiratory compromise, his pneumococcal pneumonia along with his low platelet count, blood pressure > 150 mmHg and pulse rate > 100 beats/minute, low potassium level, and low magnesium level might explain his rapid development, because these factors have been shown to predict the development of DT [11–13]. Additionally, kindling, a process of sensitization of the nervous system and heightened neuronal excitability that occurs due to multiple episodes of withdrawal, may have played an important factor in the progression of DT in our patient [1]. Although not yet endorsed at our institution, screening tools such as the Prediction of Alcohol Withdrawal Severity Scale for identifying patients at risk of developing severe alcohol withdrawal syndrome might have provided benefit in guiding prophylaxis or a treatment protocol [14, 15].

## Conclusions

The prevalence of alcohol dependence in hospitalized patients has been shown to be about 16–26% and up to 42% in hospitalized veterans [16, 17]. Approximately 5–10% of patients undergoing alcohol withdrawal progress to DT with a mortality rate of 5%, but this has been reported to be as high as 13% [17–19]. Our patient had a well-known history of alcohol abuse. Although he was being treated with the standard protocols for alcohol withdrawal, he rapidly developed DT, which led to an acute inferior STEMI in the setting of nonoccluded coronary vessels. This case report adds to the sparse literature documenting acute coronary events in the setting of alcohol withdrawal and suggests that our patient’s STEMI is not fully explained by the current coronary vasospasm hypothesis, but rather was in part the result of direct catecholamine-associated myocardial injury. As the literature grows regarding this topic, we propose that further research be conducted on prophylactic agents such as β-blockers and calcium channel blockers. High-dose β-blockers may address the underlying hyperadrenergic surge that occurs during DT and might prevent direct catecholamine myocardial injury. Calcium channel blockers are the first-line treatment for coronary artery vasospasm such as Prinzmetal angina. Their ability to promote vasodilation and increase blood flow may have a role in preventing acute coronary events during severe alcohol withdrawal if induced by an underlying vasospastic mechanism.

## Data Availability

Not applicable.

## Abbreviations

CAP: Community-acquired pneumonia
CIWA-Ar: Clinical Institute Withdrawal Assessment for Alcohol–Revised
DT: Delirium tremens
ECG: Electrocardiography
LAD: Left anterior descending artery
RCA: Right coronary artery
STEMI: ST-elevation myocardial infarction
